# Editorial: COVID-19 mechanisms on cardio-vascular dysfunction: from membrane receptors to immune response, volume II

**DOI:** 10.3389/fcvm.2023.1278067

**Published:** 2023-10-13

**Authors:** Ana Iochabel Soares Moretti, Roberto Schreiber, Amarylis B. A. Wanschel

**Affiliations:** ^1^ Laboratório de Imunologia, Instituto do Coração (InCor), LIM19, Hospital das Clínicas da Faculdade de Medicina da Universidade de São Paulo, (HCFMUSP), São Paulo, Brazil; ^2^ Department of Internal Medicine, School of Medical Sciences, State University of Campinas (UNICAMP), São Paulo, Brazil; ^3^ Department of Basic Pharmaceutical Sciences, Fred Wilson School of Pharmacy, High Point University, High Point, NC, United States

**Keywords:** LDL-receptor, COVID-19, myocarditis, NFkapapB, arrhythima, ACE-2 receptor, SARS- coV- 2, ICAM -1

**Editorial on the Research Topic** COVID-19 Mechanisms on Cardio-Vascular Dysfunction: From membrane receptors to immune response, Volume II

## Introduction

This second edition of the Research Topic “COVID-19 Mechanisms on Cardio-Vascular Dysfunction: From membrane receptors to immune response” reiterates our commitment to fill some aspects of this research lacuna by searching for original advanced and contemporary knowledge on this theme/issue. In this edition we present three relevant manuscripts: an original research article, a case report, and a review, which are summarized in this Editorial.

**Figure 1:**
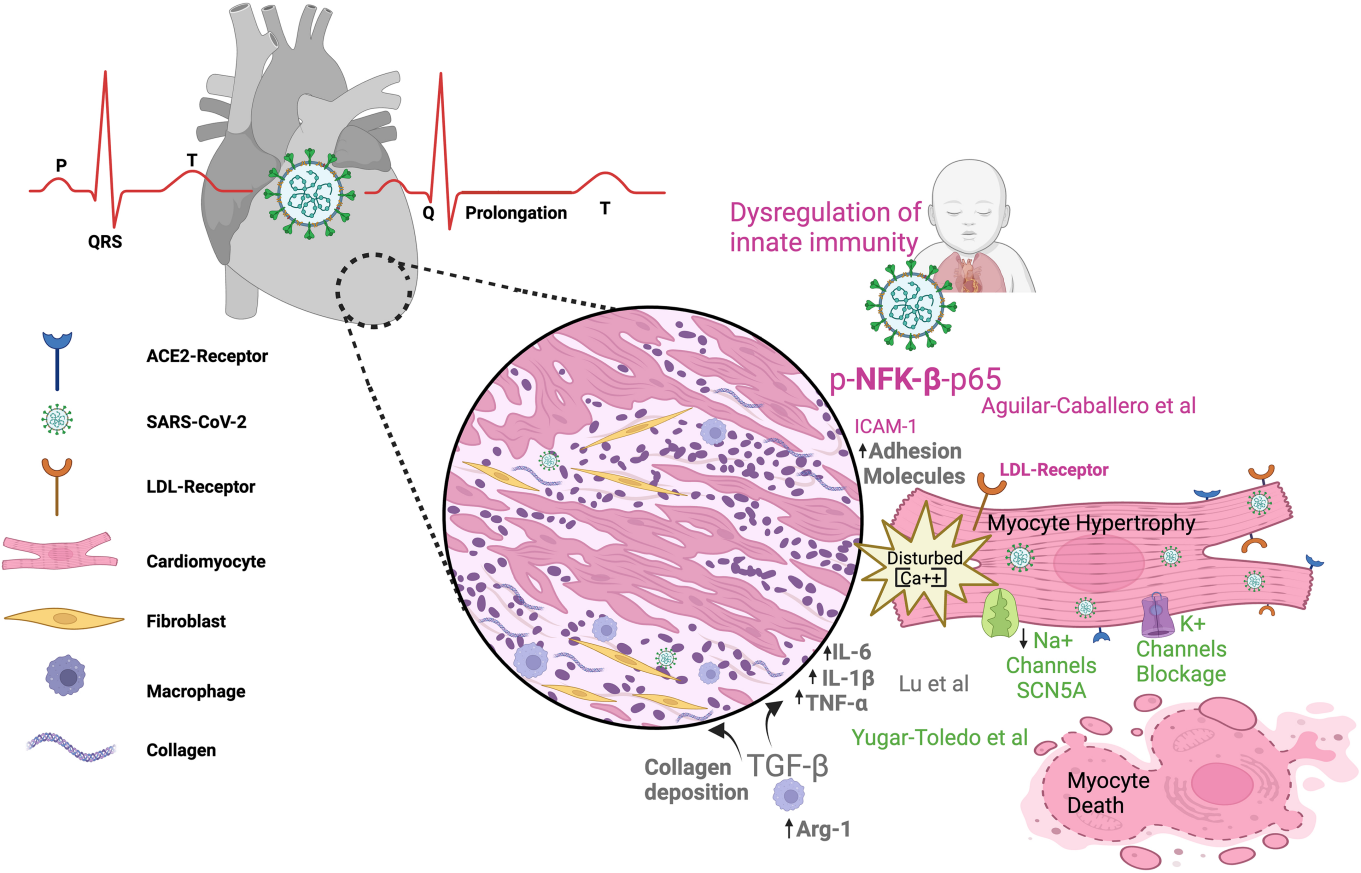
SARS-CoV-2 infection can impact the cardiovascular system in both children and adults. The figure illustrates electrocardiogram *P*, *Q*, *R*, *S*, *T* waves and their alterations in SARS-CoV-2 infection; dysregulated metabolism; inflammation; and immunity as components of the link between COVID-19 infection and heart damage. ACE2, angiotensin-converting enzyme 2; Arg-1, arginase 1; LDL, low-density lipoprotein receptor; TNFα, tumor necrosis factor alpha; (IL)-6, (IL)-1β, IL, interleukin; TGF-β, transforming growth factor–beta; NFK-β, nuclear factor kappa *B*.

Since 2020, when the World Health Organization (WHO) declared a worldwide pandemic in relation to COVID-19, it has been known that several comorbidities influence patients' outcomes, with varying degrees of impact on the severity of the disease. Nevertheless, the cause–effect relationships between molecular/cellular mechanisms and clinical manifestations remain poorly understood. In this context, cardiovascular manifestations independent of or dependent on pre-existing diseases are associated with greater severity and poor prognosis in COVID-19 patients (Figure 1).

Growing evidence indicates changes in gene signature in association with phenotype manifestations of pathophysiological conditions. Recent progress regarding the ways in which COVID-19 contributes to cardiovascular complications is assessed by Lu et al. Using bioinformatic analysis, the authors demonstrate how COVID-19 and atrial fibrillation (AF) are potentially associated with the gene signature. Transcriptome analyses unveil 54 shared differentially expressed genes (DEGs) between COVID-19 and AF, of which 34 are upregulated and 20 are downregulated genes. Among these shared DEGs, 10 key genes may become potential biomarkers and/or therapeutic targets (RPS8, BMP4, SFN, TYMS, NOG, AK5, WNT11, RLN, ARG1, and ACSL). The relationship between COVID-19 and AF includes regulatory mechanisms such as transcriptional factors, miRNAs, and drugs. Additionally, three genes (ARG1, GIMAP7, and RFX2) emerge as severity/prognosis markers linking COVID-19 to AF development. Similar studies (1, 2) have shown that, during COVID-19 disease, concomitant complications such as hypertension, heart failure, renal diseases, and diabetes mellitus type 2 share some genes and/or regulatory molecules with AF/COVID-19, suggesting that some mechanisms resulting from SARS-CoV-2 infection are common between different preexisting comorbidities. Apparently, some genes involved in negative complications that link comorbidities to COVID-19 symptoms and prognosis are not related to whether the disease onset occurred before or post SARS-CoV-2 infection.

Lu et al. also describe how the mechanisms by which COVID-19 triggers AF are associated with predominant DEGs that cause disturbances in signaling pathways related to cell metabolism, inflammation, and immunity. The emerging principles that link cellular energy disturbances and deregulated immune/inflammatory systems to disease progression are not new. Immunometabolism relationships contribute to the progression and severity of both COVID-19 (3, 4) and cardiovascular diseases (5) (Raghavan et al.), independently of their association. The cytokine release and immune cell differentiation that occurs during COVID-19 is under metabolic control (3, 4), and an understanding of how the immune response depends on intricate metabolic cell pathways is crucial in order to improve patient outcomes.

The lethality of pulmonary hyperinflammation in a preterm newborn (28 weeks) after intrauterine transmission of SARS-CoV-2 is the focus of the case report by Aguilar-Caballero et al. The authors report progressive and irreversible worsening lung hyperinflammation mediated by an imbalance of the immune response and inflammatory mediators, leading to severe and systemic clinical complications culminating in death. Elevated expression levels were observed in lung and heart detection of biomarkers related to hyperinflammation (LDLr, phospho-NF-κβ-p65, OPN), vascular dysfunction (ICAM-1), and NETs (citH3). The OPN molecule is expressed not only by immune cells but also in the vascular cells (EC and SMC) and cardiomyocytes, and circulating OPN levels predict outcomes and mechanical ventilation exigency in both adults (6) and children (7). Extreme prematurity (less than 28 weeks) is not associated with a severe course and/or poor prognosis of COVID-19 (8). Importantly, this premature infant suffered from long COVID-19 symptoms, with persistent infection consistent with the disease severity. Chronic and persistent immune dysregulation, together with an active viral infection, prevented all efforts to treat the patient and avoid progression of disease severity (9).

The review by by Yugar-Toledo et al. summarizes the main aspects of cardiovascular infection, including the clinical symptoms, during COVID-19 and sheds light on previously established comorbidities and their association with disease progression. The authors review SARS-CoV-2 damage in the myocardium, demonstrating the dependence of the inflammatory response and the three main pathways responsible for cardiac disease. These three mechanisms can affect the myocardium through the direct impact of viral entrance, through consequential damage (through down- and up-regulation of ACE2 and AT1 receptor expression, respectively), and finally, through indirect action in the form of recruitment and activation of immune cells mediating the systemic inflammatory response. The relevant consequences for the cardiovascular system include Kawasaki-like disease in children and acute coronary artery disease, arrhythmias, Takotsubo syndrome, and other cardiovascular and pulmonary complications in adults. The molecular mechanisms of cardiac arrythmias, ECG alterations, and associated findings are also reviewed, as shown by imaging assessments of cardiovascular comorbidities.

The collection of articles presented in this second special issue represents the landscape of the state of the art in cardiovascular diseases and their relationship to SARS-CoV-2 infection and COVID-19 development. In conclusion, this Research Topic highlights a two-way road connecting cardiovascular diseases and COVID-19: cardiovascular complications may be preexisting factors or consequences that aggravate COVID symptoms and prognosis. DEGs, transcriptional factors, drugs, and molecular routes emerge as a promising therapeutic targets, biomarkers for assessment of severity, and drivers of drug administration. Independently of the causal relationships, the improvement of our understanding of cardiovascular dysfunction and its underlying mechanisms will contribute to our understanding of COVID-19 pathology, progression, and prediction of clinical outcomes.
